# FC-99 ameliorates sepsis-induced liver dysfunction by modulating monocyte/macrophage differentiation via Let-7a related monocytes apoptosis

**DOI:** 10.18632/oncotarget.24127

**Published:** 2018-01-10

**Authors:** Yarong Zhao, Haiyan Zhu, Haining Wang, Liang Ding, Lizhi Xu, Dai Chen, Sunan Shen, Yayi Hou, Huan Dou

**Affiliations:** ^1^ The State Key Laboratory of Pharmaceutical Biotechnology, Division of Immunology, Medical School, Nanjing University, Nanjing, PR China; ^2^ Jiangsu Key Laboratory of Molecular Medicine, Nanjing University, Nanjing, PR China; ^3^ Novel Bioinformatics Co., Ltd, Shanghai, PR China

**Keywords:** FC-99, sepsis-induced liver injury, monocyte apoptosis, monocyte-to-macrophage differentiation, let-7a-5p

## Abstract

**Background:**

The liver is a vital target for sepsis-related injury, leading to inflammatory pathogenesis, multiple organ dysfunction and high mortality rates. Monocyte-derived macrophage transformations are key events in hepatic inflammation. N^1^-[(4-methoxy)methyl]-4-methyl-1,2-benzenediamine (FC-99) previously displayed therapeutic potential on experimental sepsis. However, the underlying mechanism of this protective effect is still not clear.

**Results:**

FC-99 treatment attenuated the liver dysfunction in septic mice that was accompanied with reduced numbers of pro-inflammatory Ly6C^hi^ monocytes in the peripheral blood and CD11b^+^F4/80^lo^ monocyte-derived macrophages in the liver. These effects were attributed to the FC-99-induced apoptosis of CD11b^+^ cells. In PMA-differentiated THP-1 cells, FC-99 repressed the expression of CD11b, CD14 and caspase3 and resulted in a high proportion of Annexin V^+^ cells. Moreover, let-7a-5p expression was abrogated upon CLP stimulation *in vivo*, whereas it was restored by FC-99 treatment. TargetScan analysis and luciferase assays indicated that the anti-apoptotic protein BCL-XL was targeted by let-7a-5p. BCL-XL was inhibited by FC-99 in order to induce monocyte apoptosis, leading to the impaired monocyte-to-macrophage differentiation.

**Materials and Methods:**

Murine acute liver failure was generated by caecal ligation puncture surgery after FC-99 administration; Blood samples and liver tissues were collected to determine the monocyte/macrophage subsets and the induction of apoptosis. Human acute monocytic leukemia cell line (THP-1) cells were pretreated with FC-99 followed by phorbol-12-myristate-13-acetate (PMA) stimulation, in order to induce monocyte-to-macrophage differentiation. The target of FC-99 and the mechanistic analyses were conducted by microarrays, qRT-PCR validation, TargetScan algorithms and a luciferase report assay.

**Conclusions:**

FC-99 exhibits potential therapeutic effects on CLP-induced liver dysfunction by restoring let-7a-5p levels.

## INTRODUCTION

Sepsis is characterized by life-threatening organ dysfunction that is caused by a dysregulated host response to infection. A total of 31.5 million sepsis cases occur every year that result to 5.3 million deaths annually worldwide [1–3]. During microbial infection, the liver is intensively exposed to circulating antigens, endotoxins, cellular signals and even microorganisms, which are transported in the blood stream either from the gut lumen via the portal vein, and/or from the systemic circulation via the arterial blood. The hepatocytes are primed to recruit additional cells to the liver in order to protect the body from the invading microorganisms [4]. Thus, the liver triggers the inflammatory response of the body in order to eliminate microorganisms efficiently, which represents a double-edged sword that results in liver damage due to an overwhelming systemic inflammatory response [5]. Liver dysfunction occurs during early sepsis that has been demonstrated at 1.5 h post CLP following mouse surgery [6, 7]. This process is an independent factor for multiple organ disorders and sepsis-induced death [5].

Monocytes and macrophages that originate from a common myeloid precursor, serve as the first line of host defense and play an important role in triggering inflammatory responses during sepsis [8]. Following liver injury, a high number of blood monocytes are rapidly recruited to the site of injury in combination with other innate myeloid cells. These cells recognize the release of foreign antigens and respond to the infection by triggering an immune-inflammatory response in order to remove the pathogens [9]. Recent studies have suggested that circulation-derived Ly6C^hi^ inflammatory monocytes infiltrate the injured liver and contribute to inflammation and the propagation of tissue damage, followed by an increase of differentiation into F4/80^lo^ tissue monocyte-derived macrophages at the early stages of liver injury [10–12]. In sepsis-induced liver dysfunction, monocytes and macrophages play central roles in the initiation and resolution of inflammation. This is facilitated via various cellular processes, namely phagocytosis, the release of inflammatory cytokines, the release of reactive oxygen species and the activation of the acquired immune system [10]. Under normal circumstances, infiltrated monocytes exhibit a short half–life and undergo spontaneous apoptosis [13, 14]. In response to differentiation factors, monocytes escape the induction of apoptosis by differentiating into macrophages with a prolonged lifespan [15]. Clinical studies revealed that the dysregulation of monocyte lifespan contributes to the pathophysiology of sepsis [16]. In experimental models of acute liver failure, massive expansions in the number of hepatic macrophages are noted within 12 h of injury. This is partly due to the recruitment of immune cells from the circulating pool of monocytes [11]. Thus, the balance of the ratio of the number of monocytes to macrophages can be used as a potential therapeutic target for the treatment of liver injury [15, 17].

Certain miRNAs have been found to play various roles in cell apoptosis by regulating the expression levels of specific proteins such as the survival factors (Bcl-2 family) that prevent apoptosis. This pathway can in turn reduce multiple-organ injury in septic mice [18, 19]. The expression of microRNA-Let7A (let-7a) has been confirmed to be significantly downregulated in gram-negative bacilli uro sepsis patients compared with healthy controls. Let-7a has been shown to regulate Toll-like receptor-mediated inflammatory response in sepsis, thereby providing a potential target for the treatment of sepsis [20]. Moreover, the overexpression of let-7a in acute myelogenous leukemia (AML) cell lines and human hepatocellular carcinoma cells resulted in the induction of apoptosis, leading to the repressed expression of the anti-apoptotic protein BCL-XL both *in vitro* and *in vivo* [21, 22]. This study demonstrated that the human miRNA let-7a negatively regulated *BCL-XL* expression in human AML cells, whereas let-7a-overexpressing cells exhibited a higher than 2-fold increase in the induction of apoptosis compared with the control cells [22].

FC-99 is a novel 1,2-benzenediamine derivative that has been reported to reduce the disarrangement of hepatocytes and morphological alterations of their nuclei in the septic liver [23]. It further suppressed the activation of NF-κB signaling [24], which was associated with a variety of responses related to inflammation, cell survival, differentiation, and proliferation [25]. However, the potential of FC-99 in the treatment of sepsis-induced liver damage has not been investigated to date. In the present study, the effect of FC-99 on liver injury was investigated in a CLP mouse model. The contribution of FC-99 in the lifespan and immune response of monocyte/macrophage subsets was evaluated. Furthermore, the effects of FC-99 on let-7a-5p/BCL-XL levels, the analysis of monocyte apoptosis, their differentiation and the extent of inflammatory injury in the liver, were investigated.

## RESULTS

### FC-99 attenuated the liver dysfunction in CLP-induced model of sepsis

**Figure 1:**
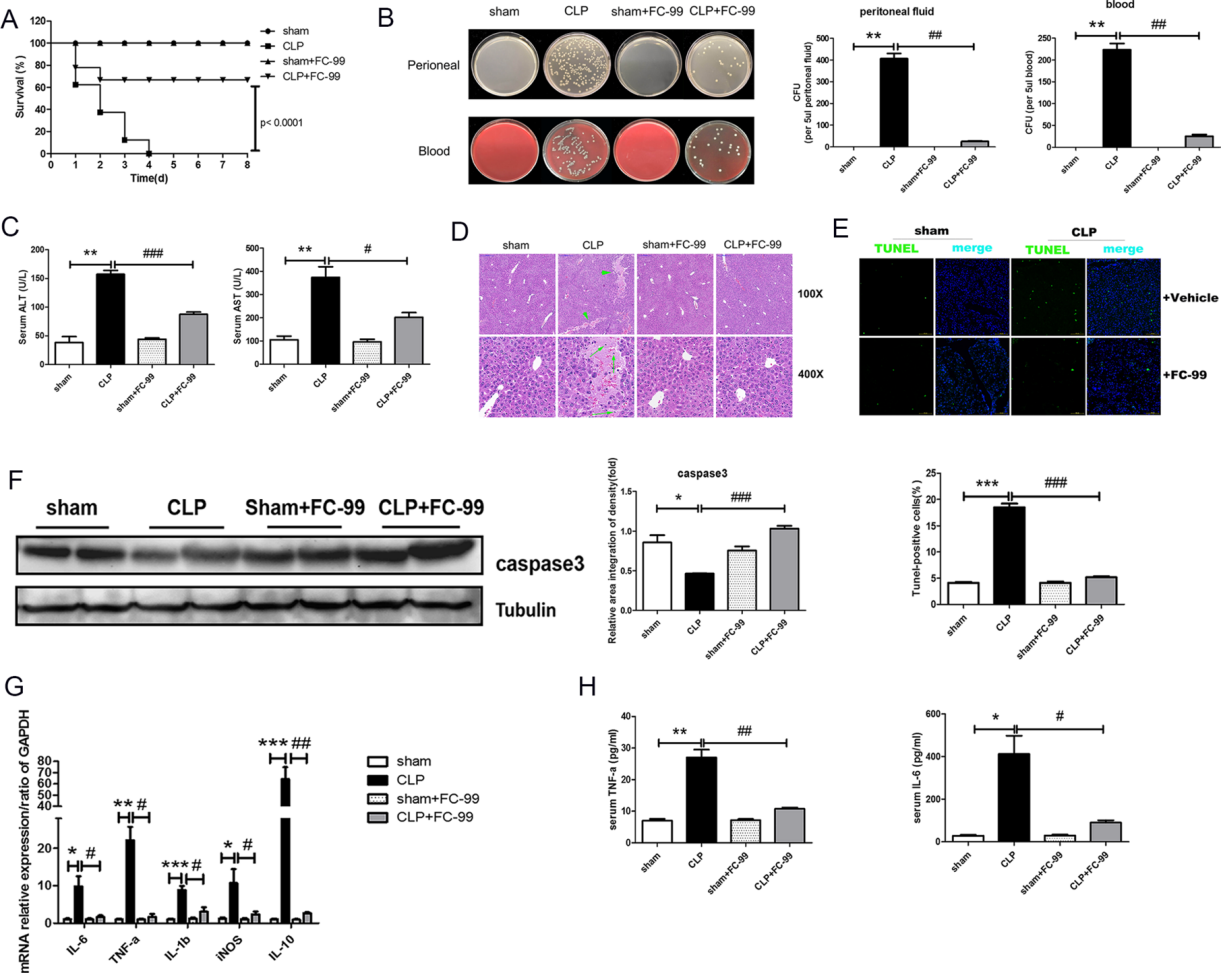
Effects of FC-99 on CLP-induced pathological changes in the liver tissue of mice All mice were pretreated with FC-99 (10 mg/kg, i.p.) 2 h prior to sham operation (sham+FC-99) and/or prior to CLP operation (CLP+FC-99), and/or prior to direct operation (sham, CLP). (**A**) The survival rate was monitored once every 24 h for 8 days, *n* = 8 miceper group). (**B**) The bacterial burden was determined by counting the number of CFUs on blood agar plates and/or LB nutrient agar plates following serial dilution of blood and peritoneal lavage samples. Blood and lavage fluid were collected at 24 h following CLP. (**C**) Serum concentrations of ALT and AST were assessed 24 h post-surgery. (**D**) H&E staining of liver tissues from each group at 24 h, as determined by a two-dimensional graph. Scale bar, 200 and/or 50 μm (magnification: upper: 100x, lower: 400x); (**E**) Hepatocyte apoptosis was detected by TUNEL assay, which displayed green immunofluorescence at 24 h (magnification 200x). Scale bar, 100 μm. Vehicle represents the non-treatment group. The histogram indicates the ratio of TUNEL-positive cells at 24 h. The data are presented as mean ± SD (*n* = 3); (**F**) Western blot analysis indicated the protein expression of caspase3 in the liver tissues; G. The mRNA expression levels of the macrophage-associated inflammatory cytokines, IL-6, TNF-α, IL-1β, iNOS, and IL-10, in the liver of CLP mice were detected by qRT-PCR. The samples were collected at 24 h post-surgery. H. The levels of IL-6 and TNF-α in the serum were determined by ELISA at 24 h. The data are presented as mean ± SEM of at least three independent experiments. ^*^P < 0.05, ^**^P < 0.01, ^***^P < 0.005, *vs.* sham operation group; ^#^*P* < 0.05, ^##^*P* < 0.01, ^###^*P* < 0.005, *vs.* CLP group; ns: no significant difference.

Our pilot -experiment indicated that the optimal time point for the assessment of the relevant liver indicators was 24 h post-CLP surgery (Supplementary Figure 1). To further investigate the role of FC-99 and its potential therapeutic effect on liver dysfunction, we established the liver injury model by inducing multi-organ injury via sepsis. In the CLP model group, mice displayed 100% mortality at 7 days, whereas, in the 10 mg/kg FC-99 treatment groups, the mortality rate was 32.5%, which indicated an effective improvement of the animal survival (*P* < 0.001) (Figure 1A). Furthermore, the bacterial colony forming unit (CFU) counts in the indicated groups of mice were measured in order to determine whether FC-99 alters the level of bacterial burden in the peritoneal lavage fluid and blood [31], which were collected 24 hours following the CLP operation. The results indicated that the bacterial CFU counts in the peritoneal lavage fluid and blood of the septic mice were significantly increased (*p* < 0.005) compared with the sham-operated mice. In contrast to the septic mice, the bacterial CFU counts of the FC-99 treated mice were significantly reduced (*p* < 0.005) (Figure 1B).

The levels of the predominant hepatocyte enzymes, also known as indicators of hepatic dysfunction, namely serum alanine transaminase (ALT) and aspartate aminotransferase (AST), were tested in order to determine the effect of FC-99 on sepsis-induced liver dysfunction [32]. The results indicated that ALT and AST levels were significantly reduced by FC-99 treatment (*p* < 0.05) (Figure 1C). The liver histological analysis for the direct observation of pathological liver changes was conducted by H&E staining and the data were indicative of extensive inflammatory infiltration and large areas of necrosis in the liver at 24 h post-CLP surgery. In contrast to the CLP mice, mice that were pretreated with FC-99 exhibited minor liver damage (long arrows indicated neutrophils and lymphocytes, short arrows represented hepatocyte necrotic area) (Figure 1D). The results were further confirmed by the assessment of cell death using the TUNEL assay, which demonstrated that FC-99 pretreatment could reduce the DNA fragmentation and cellular toxicity, and maintain the normal cellular structure and function (Figure 1E). The ratio of TUNEL-positive cells is further shown in Figure 1E. In addition, the levels of the apoptotic marker, caspase3 were determined. As expected, the protein expression indicated a similar result (*p* < 0.05) (Figure 1F). Furthermore, FC-99 reduced the levels of mRNA expression of the monocyte/macrophage-associated inflammatory cytokines, namely *IL-6, TNF-α, IL-1β, iNOS, IL-10* in the post-CLP surgery liver tissues. The findings suggested that the ratio of monocytes to macrophages may be associated with liver inflammation that could be inhibited by FC-99 treatment (*p* < 0.05) (Figure 1G). The ELISA assay indicated that FC-99 administration significantly reduced the serum levels of TNF-α and IL-6 at the indicated time periods (TNF-α, *p* < 0.001 and IL-6, *p* < 0.05) (Figure 1H).

### FC-99 inhibited the infiltration and differentiation of monocytes in the liver tissues of septic mice

**Figure 2:**
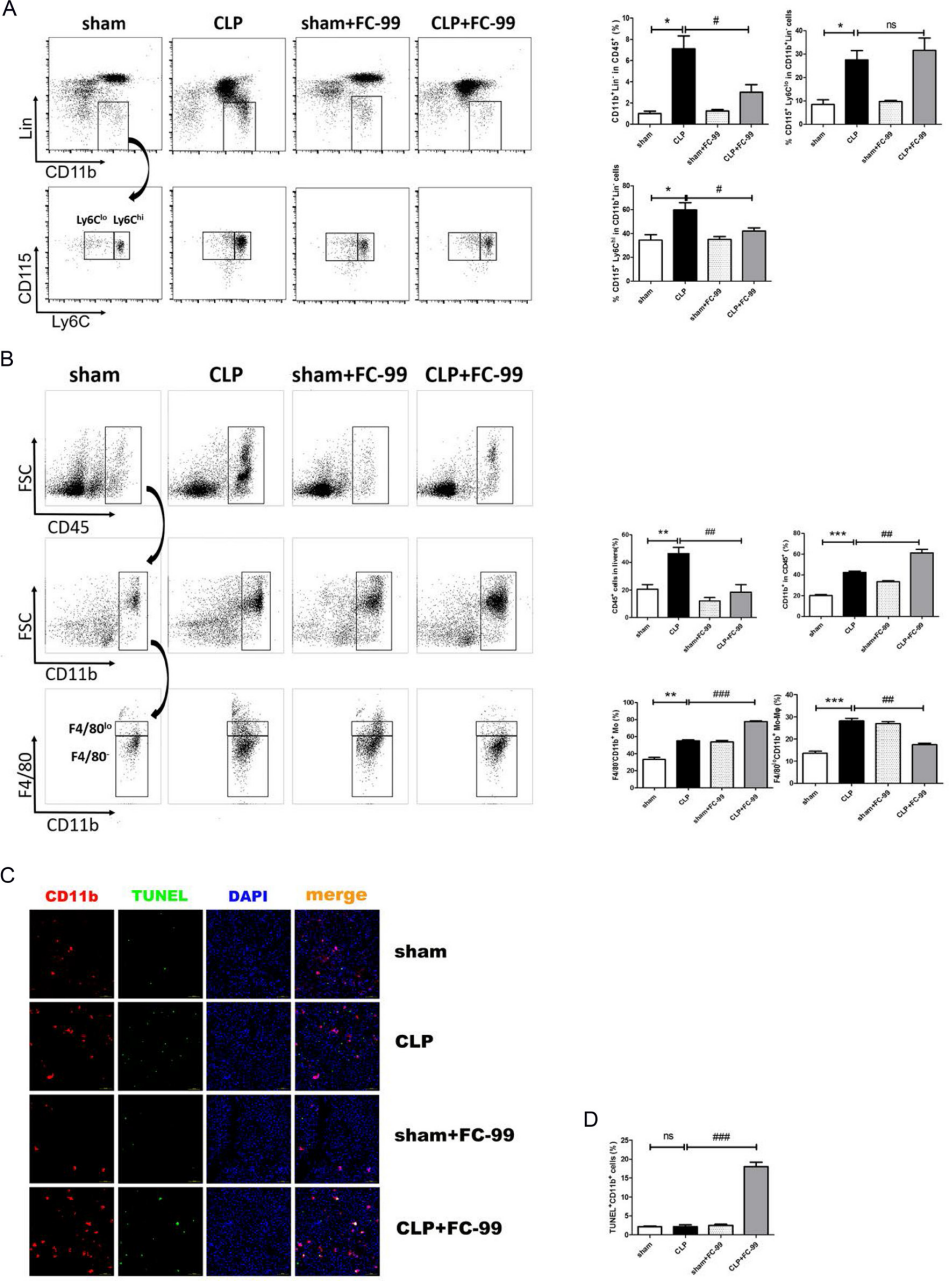
FC-99 inhibited the infiltration and differentiation of monocytes in the liver tissues of septic mice All mice were pretreated with FC-99 (10 mg/kgi.p.) 2 h prior to sham operation (sham+FC-99) and/or prior to CLP operation (CLP+FC-99), and/or prior to direct operation (sham, CLP), *n* = 8. (**A**) Flow cytometry analysis of the percentage of total monocytes (Lin^-^CD11b^+^) and their subtypes (Ly6C^lo/hi^) in the peripheral blood of each group 24 h following CLP and/or sham surgery. (**B**) Flow cytometry analysis of the percentage of lymphocytes (CD45^+^ cells), total myeloid cells (CD45^+^CD11b^+^ cells), monocyte-derived macrophages (CD45^+^CD11b^+^F4/80^lo^) and monocytes (CD45^+^CD11b^+^F4/80^-^) in the liver tissues of each group at 24 h. (**C**) Immunofluorescence detection of the apoptotic effects of FC-99 in CD11b^+^ cells in the liver tissues at 24 h post-surgery (magnification 200x). Scale bar, 100 μm. (**D**) The histogram data of apoptotic CD11b^+^ cells; ^*^P < 0.05, ^**^P < 0.01, ^***^P < 0.005, *vs.* sham operation group; ^#^*P* < 0.05, ^##^*P* < 0.01, *vs.* CLP group; ns: no significant difference.

The initial analysis on the detection of inflammatory factors in the liver such as TNF-ɑ, IL-1β, IL-6, and iNOS revealed the association between hepatitis and monocyte-macrophage cells. We speculated that monocytes were involved in the development of liver injury. Furthermore, monocytes are a heterogeneous population comprised of classical monocytes (Ly6C^hi^) and non-classical monocytes (Ly6C^lo^) [33]. The total monocyte percentage (CD11b^+^Lin^-^%) in the blood was significantly reduced by FC-99 (*p* < 0.05) treatment compared with that noted at 24 h post-CLP injury in the absence of treatment (*p* < 0.05) (Figure 2A). In addition, the frequency of Ly6C^hi^ monocytes (CD115^+^Ly6C^hi^CD11b^+^Lin^-^) in the FC-99 group was significantly lower than that in the 24 h-post-CLP mice (*p* < 0.05), while the levels of the Ly6C^lo^ monocytes (CD115^+^Ly6C^lo^CD11b^+^Lin^-^) in the FC-99 group were significantly elevated (Figure 2A). Ly6C^hi^ monocytes could trigger liver cell apoptosis/necrosis, resulting in organ failure and early cell death by perpetuating a TNF-mediated pro-inflammatory immune response [34]. In contrast to these observations, Ly6C^hi^ monocytes that infiltrated the liver differentiated into macrophages during liver inflammation as demonstrated by a previous study [35]. Thus, we hypothesized that FC-99 mainly influences the action of the pro-inflammatory Ly6C^hi^ monocytes that in turn regulate the induction of liver cell apoptosis.

At 24 h post-CLP surgery, the percentages of total CD11b^+^ cells in all infiltrated lymphocytes (CD45^+^), of the monocyte-derived macrophages (CD11b^+^F4/80^lo^) and of the liver tissue-derived macrophages, were significantly different compared with the normal control group (sham).. The number of total CD11b^+^ cells and CD11b^+^ F4/80^-^ monocytes in the liver of the septic mice increased significantly following pre-treatment with FC-99 compared with the CLP group, while the percentage of CD11b^+^ F4/80^lo^ monocyte-derived macrophages in the liver reduced dramatically. Moreover, the percentage of CD45^+^ lymphocytes increased in the diseased mice and was dramatically reduced by FC-99. The flow cytometry analysis confirmed that FC-99 inhibited the differentiation of CD11b^+^ F4/80^-^ monocytes into CD11b^+^ F4/80^lo^ monocyte-derived macrophages in the liver tissue of the septic mice. The gating strategy was illustrated in Figure 2B (*p* < 0.001). Immunofluorescence assays analyzed the Cy5-injected CD11b^+^ (a normal marker of monocytes and macrophages in mice tissues) cells in the cryopreserved liver tissue. Concomitantly, the liver tissues were labeled with an FITC-conjugated-TUNEL dye in order to detect the apoptotic CD11b^+^ cells. The results indicated that the FC-99-pre-treated septic mice were predisposed with regard to the induction of excessive apoptosis in CD11b^+^ cells compared with the CLP group (*p* < 0.005). Thus, the apoptosis induced by FC-99 occurred during the elevation of the levels of CD11b in the septic liver (Figure 2C and D). The percentage of Mo/Mϕ in the blood and liver tissues at 12, 24, and 72 h, respectively, were detected by flow cytometry (data not shown). Thus, 24 h was selected as the optimal time point for subsequent experiments. The results indicated that FC-99 could exert an anti-inflammatory effect by inhibiting the production of monocyte-derived macrophages and their infiltration to the liver.

### FC-99 induced THP-1monocyte apoptosis and inhibited their differentiation into long-lived macrophages

**Figure 3:**
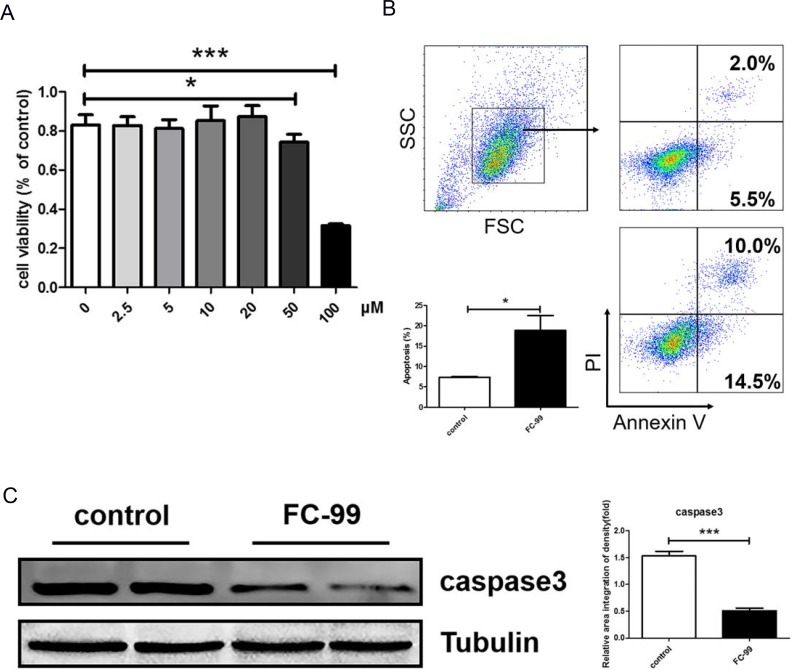
FC-99 induced THP-1 monocyte apoptosis with PMA stimulation (**A**) Cytotoxicity of FC-99 in THP-1 cells was detected by CCK-8 as described in Material and Methods. FC-99, at several indicated concentrations, was incubated with THP-1 2 h prior to treatment with PMA (2.5 ng/mL) for 48 h and detected by CCK-8. (**B**) THP-1 cells were divided into two groups: untreated group that comprised control cells treated with PMA (2.5 ng/mL) for 48 h and FC-99-treated group that comprised FC-99 (10μM) treatment 2 h prior to 48 h PMA (2.5 ng/mL) treatment). Flow cytometry with Annexin V/PI results indicated the effects of FC-99 on THP-1-induced apoptosis during differentiation. (**C**) The levels of the apoptotic marker protein, caspase 3, were examined by western blot analysis. All Results were represented as means ± SEM from four independent experiments. ^*^P < 0.05; ^**^P < 0.01; ^***^P < 0.001, vs. control group.

**Figure 4:**
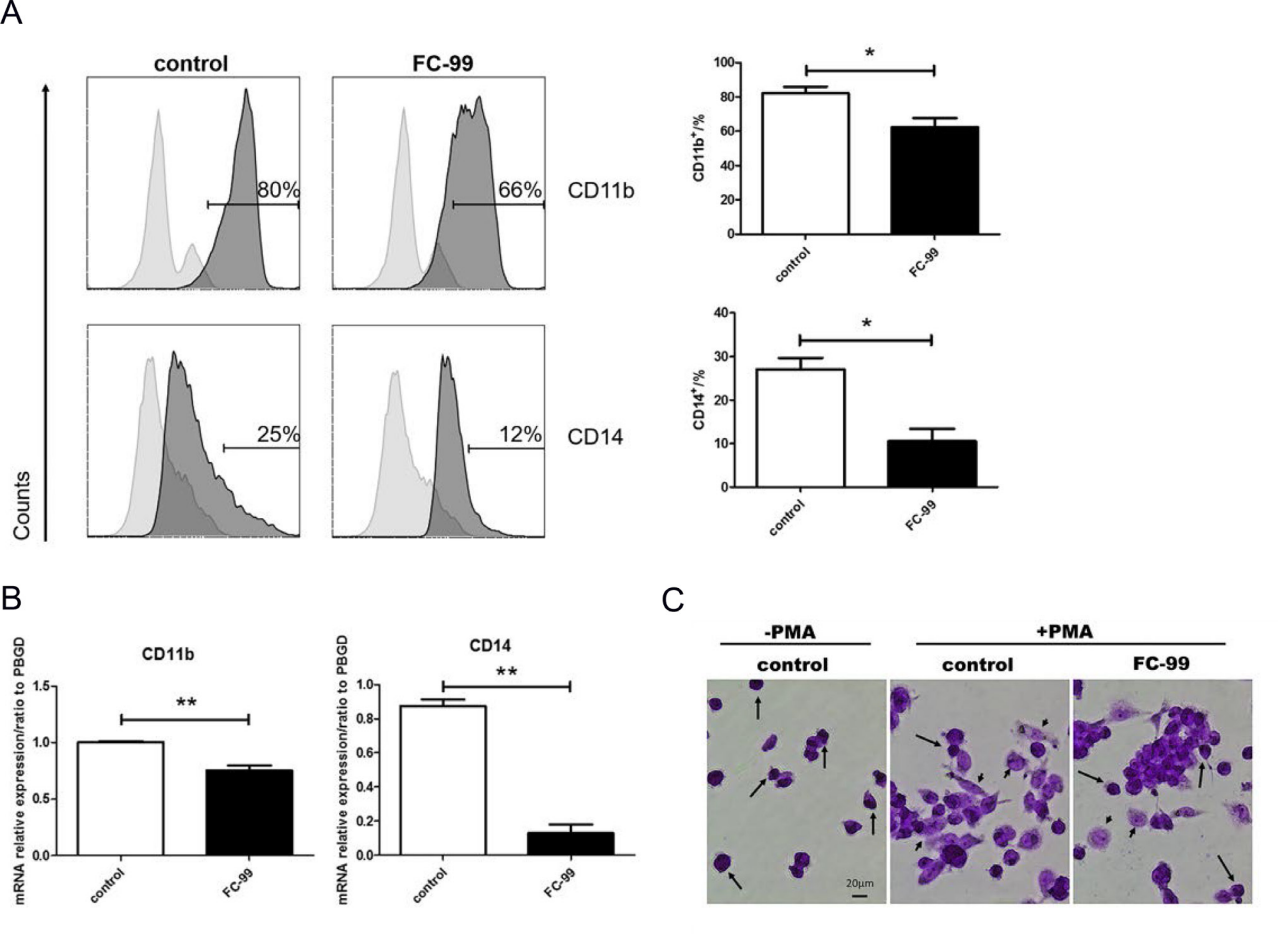
FC-99 stimulated THP-1 monocyte differentiation into long-lived macrophages THP-1 cells were divided into two groups: untreated group that comprised control cells treated with PMA (2.5 ng/mL) for 48 h] and FC-99-treated group that comprised FC-99 (10 μM) treatment 2 h prior to 48 h PMA (2.5 ng/mL) treatment. (**A**) All cells were harvested for flow cytometry analysis in order to detect the differentiation of markers, indicating the effect of FC-99 on the expression of macrophages, surface markers, CD11b and CD14, in THP-1 monocytes. (**B**) qRT-PCR displayed the expression of the differentiation-associated genes, *CD11b* and *CD14*. (**C**) Giemsa staining images were acquired using an optical microscope (original magnification, 200×), indicating the degree of monocyte differentiation into macrophages. The results were represented as means ± SEM from four independent experiments. ^*^P < 0.05; ^**^P < 0.01; ^***^P < 0.001, vs. control group.

THP-1 monocytic cells were used as a model *in vitro* system in order to assess the effective roles of FC-99 in the induction of infiltrating monocyte apoptosis and the inhibition of differentiation into macrophages in the liver tissues of the mice. The results confirmed that a concentration of 20 μM and/or lower did not reduce the cell viability for 48 h (Figure 3A). The concentration levels of FC-99 treatment that were below 20 μM were selected for further studies. In addition, the FC-99-mediated induction of CD11b^+^ monocyte apoptosis was assessed by flow cytometry in THP-1 cells. The FC-99 treatment group exhibited a significant increase in the apoptotic rate (Figure 3B) (*p* < 0.05). Moreover, the protein levels of the apoptotic marker total caspase3, were upregulated (*p* < 0.005), indicating that FC-99 promoted the induction of apoptosis (Figure 3C). In contrast to caspase3, the apparent markers used to assess the differentiation of THP-1 cells to macrophages comprised CD11b and CD14 [38]. The differentiation-associated markers (CD11b and CD14) were detected by FASC and compared to the control THP-1 cells. The data indicated that the percentage of CD11b^+^ and CD14^+^cell counts decreased dramatically in FC-99-treated cells compared with the control group (Figure 4A). The expression of the genes, *CD11b* and *CD14,* decreased at comparable levels between FC-99-treated and control groups (*p* < 0.001) (Figure 4B). In addition, Giemsa staining was applied to directly observe the effects of FC-99 in PMA-induced THP-1 differentiation. FC-99 caused an accumulation of a higher number of monocytes compared with the control group following differentiation by PMA stimulation [36] (Figure 4C). Concomitantly, the capacity of FC-99 in inducing apoptosis (Supplementary Figure 3A) and differentiation (Supplementary Figure 3B) in PMA-untreated THP-1 monocytes was analyzed. The association between let-7a-5p and monocyte differentiation was investigated in the HL-60 acute myeloid leukemia cell line and the macrophage markers CD11b and CD14 were detected by flow cytometry (Supplementary Figure 4A) and qRT-PCR (Supplementary Figure 4B).

### Let-7a-5p was increased in FC-99 induced macrophage apoptosis

**Figure 5:**
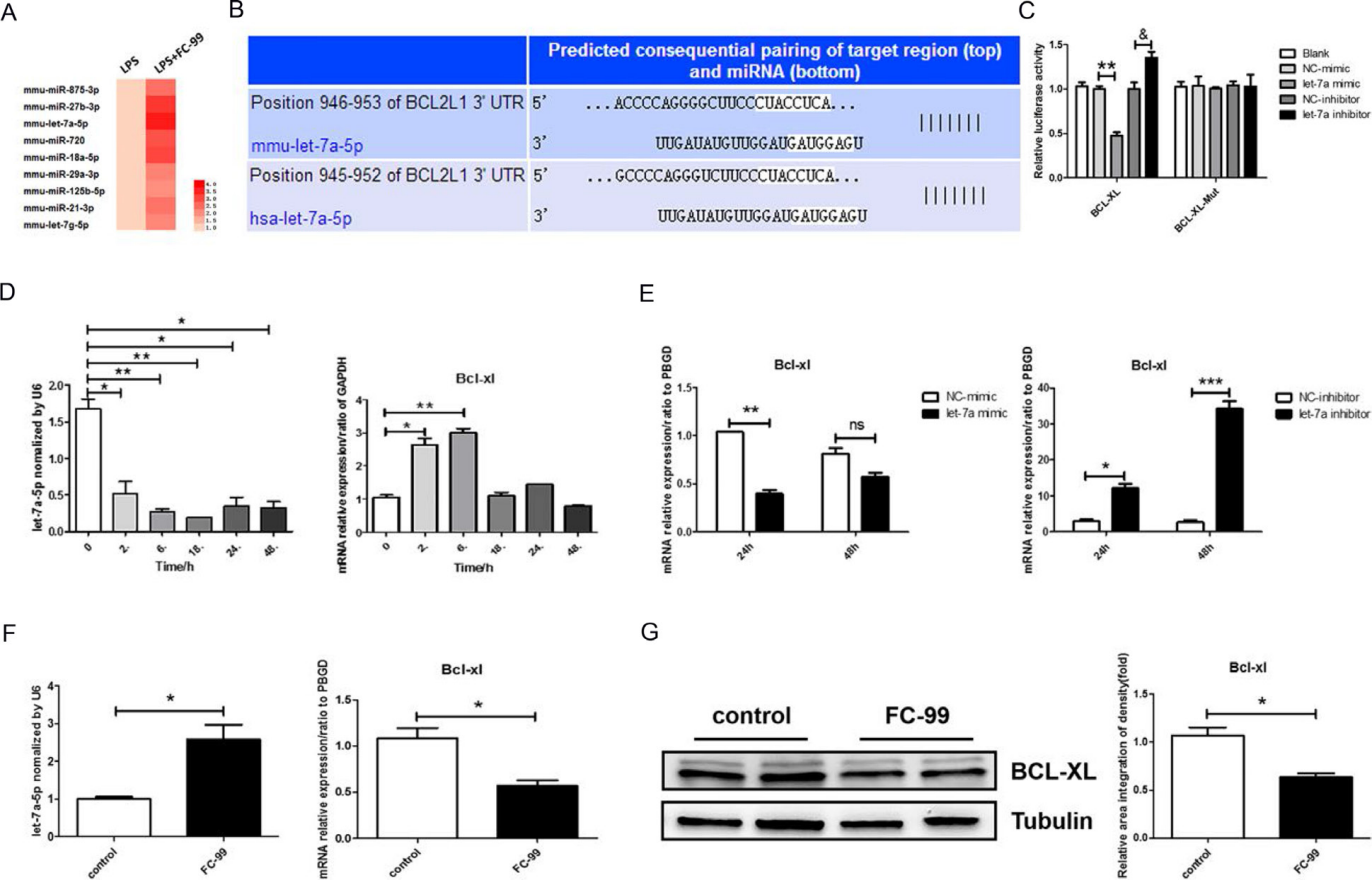
Let-7a-5p was increased during FC-99-induced macrophage apoptosis (**A**) The differences in the miRNA profile of FC-99 under LPS-simulated inflammatory stimuli were analyzed using a microarray and presented as a heatmap in which, red indicated induction of miRNA expression. (**B**) Schematic illustration of the potential let-7a-5p binding sites on the 3’-untranslated regions (3’-UTRs) of BCL2L1 (BCL-XL). (**C**) The activity of wild type and/or mutant BCL-XL-3’UTR in THP-1 cells co-transfected with control, let-7a-5p mimic, and/or let-7a-5p inhibitor as determined by luciferase reporter assays. Relative Double-luciferase activity was normalized to the scrambled oligonucleotide control. (**D** qRT-PCR assessment of the expression levels of let-7a-5p and its target gene, *BCL-XL*, in the mouse monocyte/macrophage cell line RAW264.7 at 0, 2, 6, 18, 24, and 48 h time points, respectively. (**E**) qRT-PCR evaluation of the expression of *BCL-XL* (the target gene of let-7a) in response to transfection with let-7a-5p mimic/inhibitor (50 nM final concentration) in THP-1 cells for 24 and/or 48 h. ^*^P < 0.05, ^**^P < 0.01, ^***^P < 0.005*, vs.* negative control or untreated group *in vitro*. F and G. THP-1 cells were divided into two groups: untreated group that comprised control cells treated with PMA (2.5 ng/mL) for 48 h and FC-99-treated group that comprised FC-99 (10 μM) treatment 2 h prior to 48 h PMA (2.5 ng/mL) treatment. qRT-PCR was employed to detect the expression of let-7a-5p and the mRNA levels of its target gene, *BCL-XL* (**F**). Western blot analysis with regard to the expression levels of the anti-apoptotic protein, BCL-XL (**G**). The results were represented as means ± SEM from four independent experiments. ^*^P < 0.05, vs. control group; ^**^P < 0.005, vs. NC-mimic group; &*P* < 0.05, vs. NC-inhibitor group.

miRNA has been shown to be involved in cellular apoptosis in acute liver disease by targeting key inflammatory/apoptotic proteins [18, 19]. The mechanism of FC-99-mediated protection of inflammation was examined by miRNA microarrays. The experiments aimed to detect the differential expression of miRNAs in monocytes and macrophages by comparing the samples from control RAW264.7 and LPS-stimulated RAW264.7 cells. Gram-negative bacilli infection altered the expression levels of 9 miRNAs (all were upregulated to some degree) (Figure 5A). A panel of miRNAs was selected for validation based on the statistically significant high levels of the log of fold-changes observed in the microarray. The upregulated miRNA let-7a-5p was selected for further validation by qRT-PCR. The algorithms from TargetScan (available at http://www.targetscan.org/) were used and the analysis indicated that let-7a-5p targeted the sequences in the 3’-UTR of BCL2L1 (*BCL-XL*) (Figure 5B), which ensured that *BCL-XL* was a possible target gene of let-7a-5p.

Following the identification of let-7a-5p as a main miRNA involved in Gram-negative bacilli infection, the 3’-UTR region that corresponds to the wildtype BCL-XL protein was cloned into a luciferase reporter vector in order to explore the binding of let-7a-5p on BCL-XL. This vector was transfected into THP-1 cells along with the let-7a-5p mimic and/or a negative control (NC). At 24 h post transfection, luciferase activity assays demonstrated that let-7a-5p reduced luciferase activity to 53 ± 7% in the negative control samples, whereas the mutation of the target site abolished the reduction of luciferase activity induced by let-7a-5p (Figure 5C). To further validate that let-7a-5p directly regulates the expression of BCL-XL, a let-7a-5p inhibitor and a negative control (NC) were co-transfected in THP-1 cells with separate vectors. The let-7a-5p inhibitor promoted luciferase activity to 35 ± 12% compared with the negative control transfection, while the mutant vector indicated no significant influence on luciferase activity. LPS was further used (100 ng/mL) to simulate the inflammation [37] induced in the mouse monocyte/macrophage cell line RAW264.7 and to explore the association between let-7a-5p and *BCL-XL*. The expression of let-7a-5p under LPS inflammatory simulation was reduced significantly, whereas the mRNA expression of *BCL-XL* exhibited an opposite effect following let-7a-5p addition in Mo/Mφ cells (*p* < 0.05) (Figure 5D). In addition, the target validation was essential in another mononuclear cell line, such as THP-1. The expression levels of let-7a-5p and *BCL-XL* were reversed compared with the aforementioned treatment conditions (Figure 5D and 5E). Certain studies provided clear evidence that conventional non-validated “housekeeping” genes, such as *GAPDH* and *ACTB* were not stably expressed in THP-1 differentiation experiments, and therefore, were not considered optimal for THP-1 cell models [39]. Thus, the present study used *PBGD* as a reference gene in THP-1 cells. The results indicated that the transfection efficiency was optimal at 24 h (*p* < 0.05) and as a result this time point was selected for further applications (Figure 5E). Cy3-conjugated negative control was transfected in THP-1 cells for 24 and/or 48 h, and the transfection efficiency was determined by optical microscopy (Supplementary Figure 5A). The expression of *let-7a-5p* and *BCL-XL* in transfected THP-1 cells at 24 and/or 48 h indicated that 24 h was the time point at which the maximum transfection efficiency was noted (Supplementary Figure 5B). The direct effect of FC-99 on let-7a-5p in PMA-induced monocyte differentiation was subsequently examined. FC-99 increased the miRNA expression of let-7a-5p and resulted in the reduction of its target gene, *BCL-XL* in PMA-treated THP-1 cells (*p* < 0.05) (Figure 5F). The levels of the corresponding protein expression of BCL-XL were similar to those of let-7a-5p (*p* < 0.05) (Figure 5G). In addition, the expression levels of *let-7a-5p* and *BCL-XL* in PMA-untreated THP-1 monocytes (Supplementary Figure 3C) and HL-60 (Supplementary Figure 4C) were analyzed.

Taken together, the data demonstrated that *BCL-XL* could be a target gene of miRNA let-7a-5p, and that FC-99 might exert a direct regulation on let-7a-5p under LPS or PMA stimulation *in vitro*.

### FC-99 elevated let-7a-5p expression levels and inhibited the expression of its target gene BCL-XL

**Figure 6:**
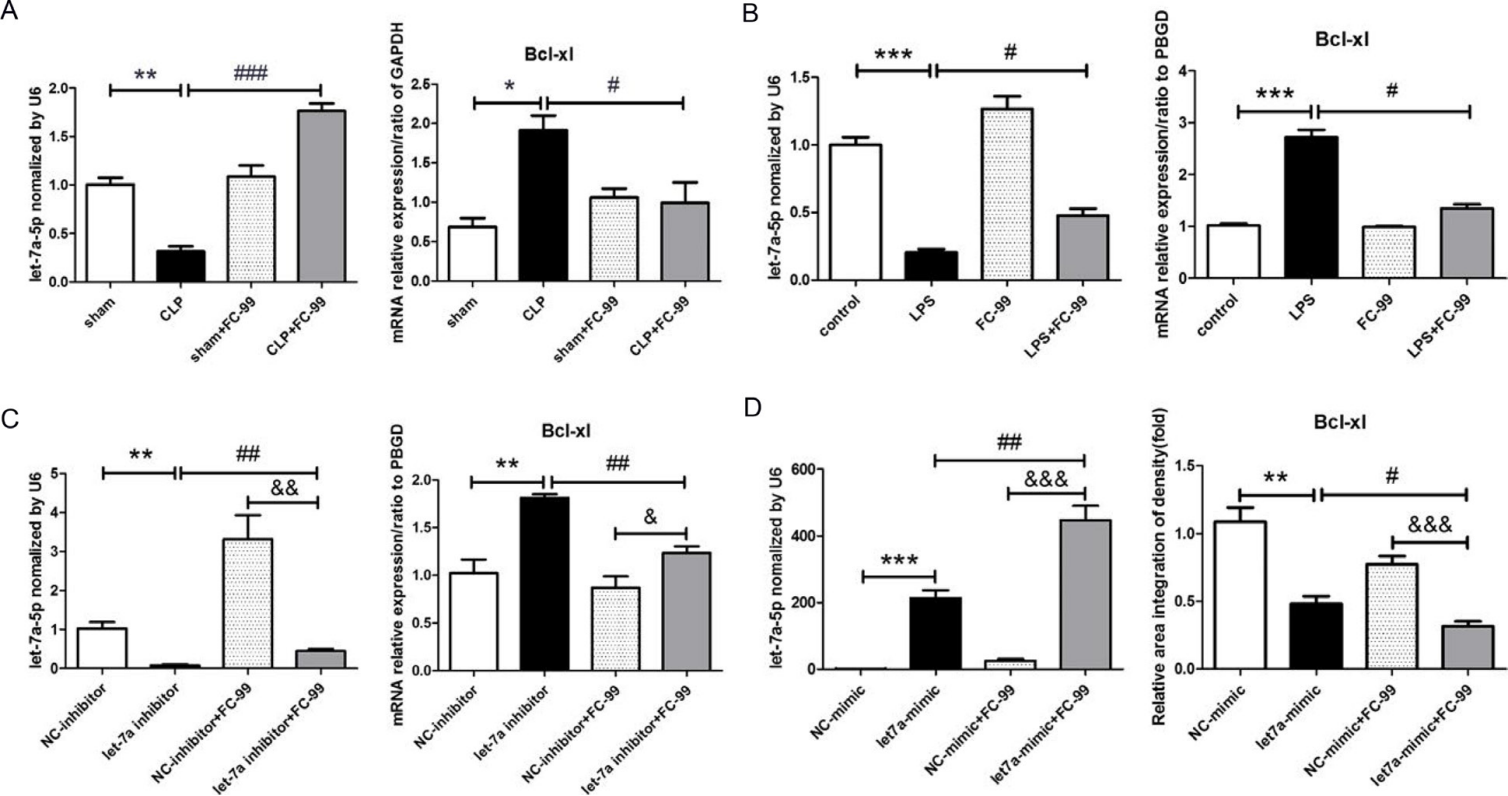
FC-99 increased the expression of let-7a-5p that inhibited the expression of its target gene BCL-XL (**A**) qRT-PCR evaluation of the expression levels of let-7a-5p and its target gene *BCL-XL* in response to FC-99 (10 mg/k, i.p.) in the septic liver at 24 h. (**B**) LPS (100 ng/mL) was applied in order to induce inflammation *in vivo*. THP-1 cells were pre-treated with FC-99 (10 μM) and/or vehicle 2 h prior to the addition of LPS for another 24 h. The expression levels of let-7a-5p and BCL-XL mRNA were detected by qRT-PCR. (**C**) THP-1 cells were transfected with let-7a-5p mimic (50 nM final concentration) for 24 h, followed by the addition of FC-99 (10 μM) for another 24 h in order to determine the effects of let-7a-5p on FC-99. (**D**) THP-1 was transfected with let-7a-5p inhibitor (50 nM final concentration) for 24 h, and subsequently with FC-99 (10 µM) for another 24 h in order to determine the effects of FC-99 on let-7a-5p. The expression levels of let-7a-5p and *BCL-XL* were assessed by qRT-PCR. ^*^P < 0.05, ^**^P < 0.01, ^***^P < 0.005, *vs.* sham operation group *in vivo* and/or *vs.* control group *in vitro*; ^#^*P* < 0.05, ^##^*P* < 0.01, ^###^ *P* < 0.005, *vs.* CLP group and/or *vs.* LPS group and/or *vs.* let-7a inhibitor group *in vitro*, &*P* < 0.05, &&*P* < 0.01, &&&*P* < 0.005, *vs.* NC-inhibitor+FC-99 group *in vitro*.

The results of qRT-PCR demonstrated that the expression of let-7a-5p in the CLP group was significantly lower than that in the sham group *(p* < 0.01), whereas the target gene of let-7a-5p, *BCL-XL*, exhibited an opposite pattern of expression (*p* < 0.05). FC-99 treatment reversed the elevated *let-7a-5p* and *BCL-XL* expression levels that were caused by CLP application (*P* < 0.005 in *let-7a-5p* and *P* < 0.05 in *BCL-XL*) (Figure 6A). Furthermore, the expression levels of *let-7a-5p* and *BCL-XL* in CLP mice at various differentiation time points (12, 24, 72 h) were examined (Supplementary Figure 2). The studies were designed to mimic the induction of inflammation by LPS treatment in order to determine the effect of FC-99 on let-7a-5p in THP-1 monocytes. Consequently, the pattern of expression of *let-7a-5p* and *BCL-XL in vitro* was similar to that noted *in vivo* (*P* < 0.05) (Figure 6B). In order to investigate the effect of FC-99 on let-7a-5p, we knocked down and/or up-regulated the expression of let-7a-5p with RNAi (Figure 6C) and/or let-7a-5p mimic, respectively (Figure 6D). The transfection experiments were conducted for 24 h in THP-1 and subsequently FC-99 was added and incubated with the cells for an additional 24 h. The interference of the expression of let-7a-5p was increased rapidly by FC-99 (*p* < 0.01). In contrast to let-7a-5p, the expression of the target gene *BCL-XL* was increased following the inhibition of let-7a-5p expression by FC-99 (*p* < 0.01) (Figure 6C). Although the expression of let-7a-5p was increased in THP-1 cells, this effect was enhanced by FC-99 treatment (*p* < 0.01). BCL-XL exhibited a decreased pattern of expression that was further promoted by FC-99 treatment (*p* < 0.05) (Figure 6D). In addition, the difference in the expression levels of let-7a-5p and BCL-XL between the FC-99 treated control group and the let-7a-5p expression group was significant (*p* < 0.05); let-7a-5p and BCL-XL exhibited an opposite pattern of expression.

### FC-99 induced monocyte apoptosis and inhibited their differentiation by up-regulating let-7a-5p

**Figure 7:**
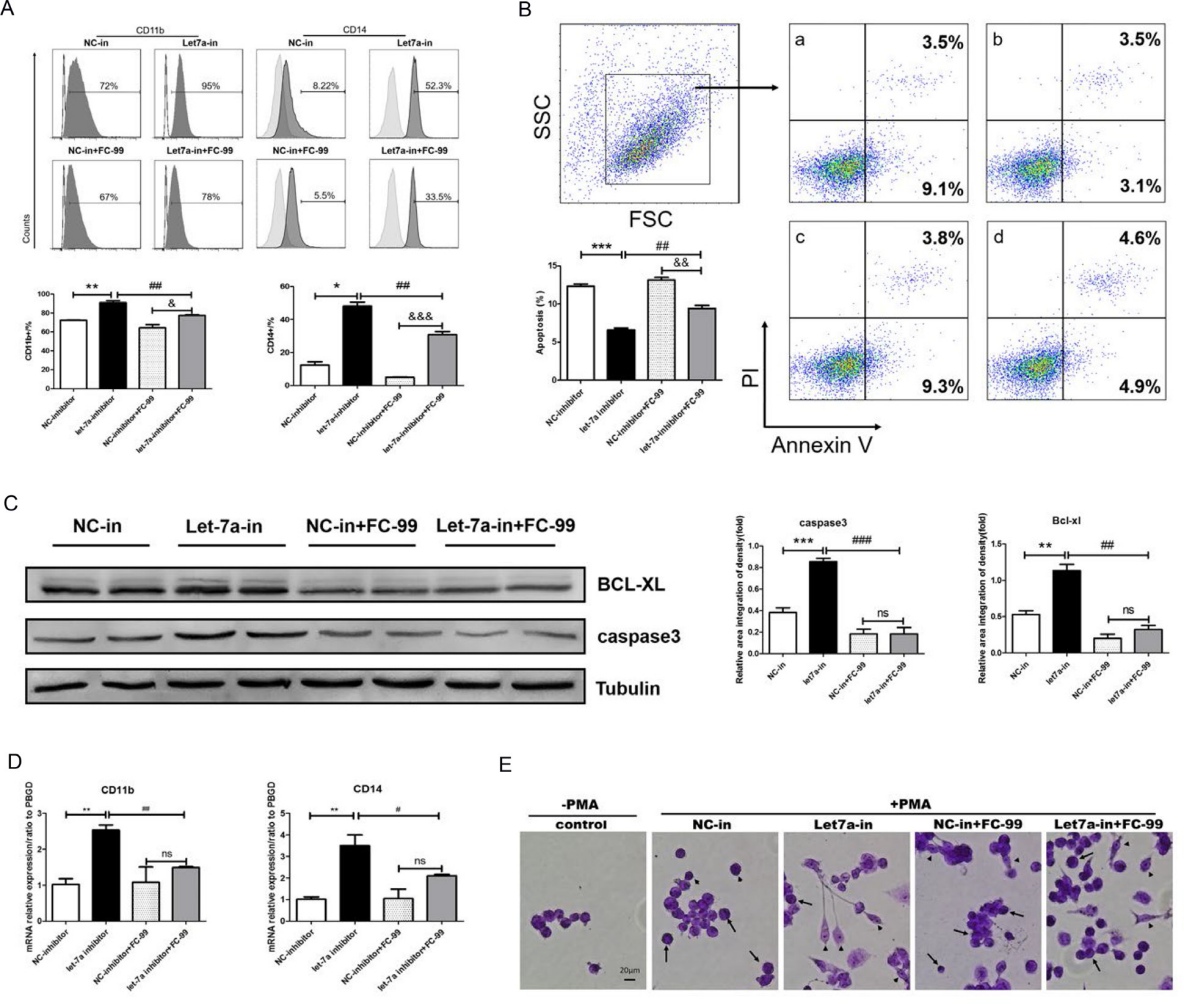
FC-99 induced monocyte apoptosis and inhibition of their differentiation by up-regulation of let-7a-5p All THP-1 monocytes in the following experiments were knocked down using the let-7a inhibitor (50 nM final concentration) for 24 h and were subsequently treated with FC-99 (10 µM) 2 h prior to 24 h incubation with PMA (2.5 ng/mL); NC-inhibitor (50 nM final concentration) was used as a control group. (**A**) All THP-1 cells were harvested for flow cytometry in order to detect the differentiation markers and the induction of apoptosis, indicating the effect of FC-99 on the expression of the macrophage surface markers, CD11b and CD14, in THP-1 monocytes. (**B**) Annexin V/PI assay detection of apoptosis in monocytes induced by FC-99.a: NC-inhibitor, b: let-7a-inhibitor, c: NC-inhibitor+FC-99, d: let-7a-inhibitor+FC-99. (**C**) Western blot analysis of the levels of the apoptotic marker caspase3 and the anti-apoptotic protein BCL-XL. (**D**) qRT-PCR indicated the mRNA expression of macrophage surface markers, *CD11b* and *CD14*, following PMA induction. E. Giemsa staining images were acquired with an optical microscope (original magnification, 200×) and indicated the degree of monocyte differentiation into macrophages. The long arrows represent the undifferentiated monocytes, whereas the short arrows indicate the differentiated macrophages. The results are represented as means ± SEM from four independent experiments. ^*^P < 0.05; ^**^P < 0.01; ^***^P < 0.001 vs. NC-inhibitor group. ^#^*P* < 0.05; ^##^*P* < 0.01; ^###^*P* < 0.005 vs let-7a-5p-inhibitor group. ^&^*P* < 0.05, ^&&^*P* < 0.01, ^&&&^*P* < 0.005, *vs.* NC-inhibitor+FC-99 group *in vitro*; ns: no significant difference.

**Figure 8:**
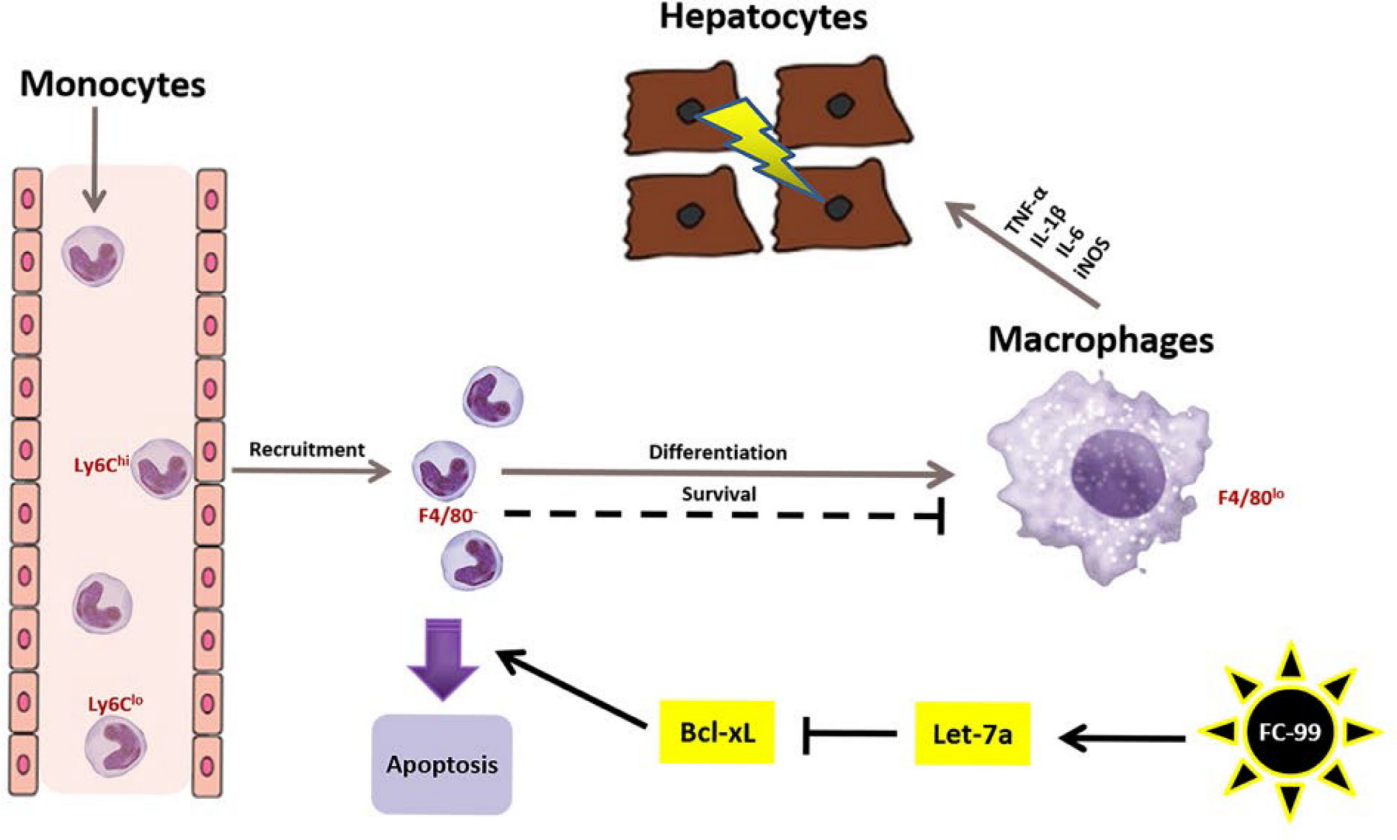
Mechanism of FC-99-mediated anti-inflammatory function in the septic liver The illustration represents the mechanism of FC-99 in relieving liver injury in mice that were challenged by CLP surgery. The brown arrows indicate the route of peripheral blood monocytes that were recruited to the liver as well as the inhibition of their differentiation into macrophages. The black arrows indicate the effects of FC-99 on mononuclear cells that result in the induction of apoptosis. The small red print represents the markers of immune cells. For example, Ly6C^hi^ and Ly6C^lo^ demonstrate the subsets of monocytes, respectively, which express the Ly6C^hi^ or Ly6C^lo^ markers, whereas F4/80^-^ and/or F4/80^+^ represent the monocytes and macrophages, respectively. The arrows indicate stimulation, while the straight lines represent inhibition.

The decline in the differentiation markers, CD11b and CD14, was detected by FACS, and the results indicated that THP-1 macrophages were decreased dramatically in the presence of the let-7a-5p inhibitor and FC-99 compared with the let-7a-5p inhibitor-treated group (*p* < 0.01) (Figure 7A). The expression of the genes *CD11b* and *CD14* was decreased at a similar level between the FC-99 and let-7a-5p inhibitor-treated cells and the let-7a-5p inhibitor-treated groups (Figure 7D). Flow cytometry (Figure 7B) and WB assays (Figure 7C) were used to assess the apoptotic markers. The data indicated that the FC-99 treatment group could significantly down-regulate the anti-apoptotic protein BCL-XL (*P* < 0.01), thereby promoting monocyte apoptosis. Moreover, THP-1 cells that were transfected with let-7a-5p mimic and/or inhibitor were subjected to Western blotting (Supplementary Figure 5C) and flow cytometry analyses (Supplementary Figure 5D) in order to assess the effects of let-7a-5p on the induction of apoptosis. In summary, the increase in the induction of monocyte apoptosis caused by FC-99 may be involved in the regulation of the expression of let-7a-5p and its target gene *BCL-XL* (*P* < 0.05) (Figure 6D). Giemsa staining was used in order to directly observe the degree of monocyte differentiation into macrophages. The let-7a-in+FC-99 group exhibited a decreased number of macrophages compared with the let-7a-inhibited-group. Long and short arrows indicated monocytes and monocyte-derived-macrophages, respectively. Let-7a-deficient THP-1 cells were susceptible to differentiation, while FC-99 could prevent THP-1 monocytes from differentiating into macrophages in the presence of PMA (Figure 7E). Thus, we hypothesized that FC-99 plays a role in inhibiting monocyte-to-macrophage differentiation by overexpressing let-7a-5p and promoting the monocyte-induced apoptosis, thereby reducing the release of inflammatory cytokines (Figure 8).

## DISCUSSION

The liver has been shown to be susceptible to sepsis-induced inflammation [40]. Consequently, the present study focused on murine poly-microbial sepsis (CLP method) in a liver tissue model in order to explore the pathophysiological characteristics of multiple organ injuries. The previous study conducted by our group described the anti-inflammatory actions of a novel benzenediamine derivate namely, FC-99 and its applications to the CLP-challenged mouse model in order to identify whether it could ameliorate the CLP-induced liver injury. As a result, FC-99 reduced the induction of apoptosis of mouse hepatocytes and cytokine production and improved their survival rate. Furthermore, the Ly6C^hi^ Mo subsets that were derived from peripheral blood were decreased. This effect blocked their differentiation into macrophages, leading to the accumulation of liver monocytes. In addition, FC-99 caused apoptosis in CD11b^+^ cells that were derived from liver tissues of CLP mice, which indicated that FC-99 protected the CLP-induced liver injury by promoting the induction of apoptosis of CD11b^+^ cells. This in turn inhibited the Mo cell conversion to macrophages and the secretion of the corresponding cytokines, thereby significantly alleviating the inflammation-mediated liver injury.

Recent studies have reported that FC-99 protects CLP mice from sepsis-induced organ injury and systemic inflammation, including liver and lung histological dysfunction, which is consistent with the results of the current *in vivo* experiment. The present study demonstrated the significant anti-inflammatory effect of FC-99. In addition, the levels of the macrophage-related cytokines *TNF-α, IL-1β, IL-6, iNOS*, and *IL-10* were increased during CLP, which suggests that liver injury could be induced by macrophages. It is widely accepted that macrophages differentiate from monocytes that are recruited from the peripheral blood. Thus, we detected the percentage of monocytes in the peripheral blood and liver tissues and the percentage of macrophages in the liver tissues. The elevated levels of monocytes and macrophages in the blood and liver tissues suggested that the incidence of liver inflammation was associated with an increase in the numbers of monocytes and macrophages. In addition their differentiation capacity was associated with the degree of liver inflammation. The monocyte-to-macrophage differentiation plays a key role in the pathophysiology of inflammation [14]. Based on this hypothesis, we examined the alterations in the mononuclear cells under FC-99 treatment conditions. It is interesting to note that the number of monocytes was increased, while the number of macrophages was reduced rapidly in the liver tissues. Thus, we speculated that the accumulation of monocytes and the reduction in the number of macrophages was inhibited due to the differentiation pathways of monocytes-to-macrophages. In contrast to these observations, the FC-99-induced apoptosis in CD11b^+^ cells, which included the infiltrated monocytes, could explain the reduction noted in the number of macrophages. Several reports revealed that a balanced network of cell survival and cell death determines the fate of monocytes [41]. Therefore, the pathway of apoptosis that was promoted by FC-99 treatment would inhibit the differentiation process of monocytes to macrophages. However, the exact mechanism by which FC-99 can cause monocyte apoptosis is yet unknown.

Previous studies further demonstrated that some miRNAs play various roles in the regulation of cellular apoptosis [42, 43]. In addition, miRNA let-7a is implicated in the induction of apoptosis [44]. Moreover, in the current study, the expression of let-7a-5p was identified as a biomarker of Gram-negative bacilli sepsis and was significantly downregulated in the peripheral leukocytes of patients with sepsis. We found that let-7a-5p had a lower expression in the liver tissues of FC-99-treated mice compared with control mice. This conclusion was also confirmed using an *in vitro* model of the human monocyte cell line, which revealed that LPS stimulation downregulated the expression of let-7a-5p in THP-1 cells. The miRNA microarray results indicated that FC-99 treatment caused a high expression of let-7a-5p. This result was further confirmed by *in vitro* cell experiments, where let-7a-5p expression was silenced, and FC-99 was added in LPS-pre-treated THP-1 cells.

In addition, LPS-induced TNF-α and IL-1β secretions were reduced following let-7a-5p overexpression in THP-1 cells [20]. Furthermore, the increased production of inflammatory cytokines, such as TNF-α and IL-6, has been widely observed in the septic liver [45]. Taken collectively, these findings suggested that let-7a-5p might be a key regulator in the aggravation of liver injury in sepsis, which requires further investigations. Thus, in the present study, the inhibition of let-7a-5p resulted in significantly increased concentrations of TNF-α, IL-1β, IL-6, and iNOS and decreased hepatic function in mice with sepsis. Although the roles of let-7a-5p in septic hepatocytes have not yet been fully elucidated, we speculated that the down-regulation of let-7a-5p could cause an aggravation of hepatic inflammation and dysfunction. Consequently, the target gene of let-7a-5p, *BCL-XL*, which is an anti-apoptotic gene, could be down-regulated by FC-99. As a result, we hypothesized that let-7a-5p could inhibit the monocyte-to-macrophage differentiation during the progression of liver injury. In addition, the overexpression of let-7a-5p in AML cells led to a reduced expression of BCL-XL and enhanced apoptosis [46]. The underlying regulatory mechanism of let-7a-5p that contributes to the progression of liver injury was examined by the correlation between let-7a-5p and BCL-XL and their effects on the monocyte-to-macrophage differentiation. In the current study, the expression of let-7a-5p was down-regulated and its efficacy was decreased following the administration of FC-99. Moreover, the differentiation of mononuclear cells to macrophages was increased, whereas the increased apoptosis was inhibited.

Several factors affect the differentiation of monocytes to macrophages. However, additional studies are required to clarify the underlying mechanisms. For example, some reports revealed that autophagy plays a major role in Mo/Mφ differentiation [47], which will be the focus of our future research.

In conclusion, the present study indicates that FC-99 reduced the serum levels of inflammatory factors, the induction of hepatocyte apoptosis, and the infiltration of monocytes in the liver, thereby implying the regulatory roles of FC-99 in monocyte differentiation via let-7a-5p. The data suggest that FC-99 can ameliorate the CLP-induced liver injury.

## MATERIALS AND METHODS

### Reagents and materials

FC-99, (purity ≥ 99%) was solubilized in dimethyl sulfoxide (DMSO; Biosharp, Hefei, Anhui, China) and diluted with saline. PMA, *Escherichia coli* lipopolysaccharide (LPS), collagenase type IV and DNase I were purchased from Sigma-Aldrich (St. Louis, MO, USA). The antibodies used for caspase3 and BCL-XL western blot detection were raised in mouse and were obtained from Cell Signaling Technologies (CST; Beverly, MA, USA), whereas the peroxidase-conjugated antibodies were goat anti-mouse IgG and goat anti-rabbit IgG, respectively and were from Thermo Scientific (Landsmeer, The Netherlands). The antibody for tubulin was also purchased from Thermo Fisher Scientific (Berlin, Germany). Anti-mouse CD3e-PE, Ter-119-PE, CD49b-PE, CD45R-PE, NK1.1-PE, CD45-FITC, F4/80-PE and CD16/32 FC-blocker antibodies were purchased from eBioscience (San Diego, CA, USA). Anti-mouse CD115-Alexa Flour 488, Ly6G-PE and anti-human CD14-FITC were obtained from Biolegend (San Diego, CA, USA). Anti-mouse Ly6C-APC was obtained from BD Pharmingen (San Diego, CA, USA) and anti-mouse/human CD11b-PE-Vio770 from Miltenyi Biotec (GmbH, Bergisch-Gladbach, Germany).

### Mice and CLP model

The handling of the animals and the experimental procedures were conducted in strict accordance with the Research Ethics Committee of Nanjing University. Male C57BL/6 mice that were 8 to 10 weeks old and weighted 25 to 30 g were obtained from Model Animal Genetics Research Center at Nanjing University (Nanjing, China). All mice were maintained under specific pathogen-free conditions and 12 h light/dark cycles.

CLP surgery is a classical experimental method used to establish a sepsis mouse model as follows: Vehicle (DMSO in normal saline) and/or FC-99 was administered (i.p.10 mg/kg) to mice 2 h prior to CLP surgery and/or sham operation [26]. For poly-microbial sepsis, mice were anesthetized with 2 g/kg ethyl carbamate by i.p. injection. Under aseptic conditions, the mice were subjected to sham and/or CLP surgery as described above. Following opening of the abdomen, the cecum was temporarily removed from the abdominal cavity with tweezers and ligated by 3 silk ligatures at its base in the absence of obstruction of the intestinal continuity. The cecum was punctured two times and squeezed to excrete the small amount of fecal material into the peritoneal cavity. The cecum was then returned into the peritoneal cavity, and the abdominal incision was closed with 3 silk ligature sutures. Following the surgical procedure, the mice were resuscitated with 1 mL pre-heated normal saline solution at 37°C by subcutaneous injection in order to replace the fluid and blood loss that was caused during the operation. The mice were placed in a supine position under a heating lamp until recovery. In addition, the animals were monitored every 12 h for a total period of 7 days following the induction of sepsis in order to study the effects of FC-99 on survival. A total of 32 mice were randomly assigned to control (sham and sham+FC-99) and experimental groups (CLP and CLP+FC-99). These two groups were divided into two subgroups that comprised 8 animals each. At 24 h post-surgery, sera and liver were collected for alanine aminotransferase (ALT) and aspartate aminotransferase (AST) measurements (Beckman Coulter, CA, USA), hematoxylin and eosin (H&E) -staining (Google Biotech, Wuhan, China), and plasma pro-inflammatory cytokine detection, namely TNF-α (Biolegend, Fell, Germany) and IL-6 (FCMACS, Nanjing, Jiangsu, China).

### Bacteria burden assay

A bacterial burden assay was conducted in order to confirm the successful application of surgery and FC-99 on the sepsis-induced liver dysfunction model. A total of 32 mice were randomly assigned to control (sham and sham+FC-99) and experimental groups (CLP and CLP+FC-99). The mice were sacrificed 24 h following CLP, and peritoneal lavage fluid and blood were collected. Following serial dilutions of peritoneal lavage and/or blood, 5 μl of each sample was transferred on LB nutrient agar plates and/or blood agar plates respectively. The bacteria were counted following incubation at 37°C for 24 h and their number was estimated as CFU per whole peritoneal lavage or blood. Each group comprised 8 mice.

### Immunofluorescence (IF)

Frozen sections of mouse liver were obtained. The sections were washed three times with phosphate-buffered saline (PBS) and blocked at 15 to 25 °C for 30 min. IF was conducted as previously described [27].

### TdT-mediated dUTP nick-end labeling (TUNEL) assay

TUNEL assay was conducted with the *in situ* cell death detection kit that contained fluorescein (Roche Molecular Biochemicals, Indianapolis, IN, USA), according to the manufacturer’s instructions.

### Flow cytometry

The analysis of intrahepatic monocytes and macrophages was conducted using liver single cell suspension that was previously subjected to density gradient centrifugation (P4937, Sigma-Aldrich, St. Louis, MO, USA) at 2,000 rpm for 30 min at 25°C. Leukocytes were collected from the interphase following centrifugation and washed twice with PBS containing 2% FBS. Subsequently, the cells were blocked with mouse CD16/32 at room temperature for 15 min, followed by staining with FITC-CD45, PE-F4/80, PE-Vio770-CD11b, and APC-Ly6C for 30 min at 4°C in the dark [28]. Following washing with PBS, the cells were analyzed on a BD ACCURI C6 flow cytometer (BD Biosciences, Ann Arbor, MI, USA). The blood monocyte subsets were incubated with Alexa 488-CD115, PE-Ly6G, PE-CD3e, PE-Ter119, PE-CD49b, PE-NK1.1, PE-CD45R, PE-Vio770-CD11b and APC-Ly6C [29].

All the cells were collected and pre-incubated with FITC-CD14 and PE-Vio770-CD11b in order to detect the induction of differentiation and apoptosis in THP-1 monocytes. Apoptosis of THP-1 cells was assayed with an Annexin V-Alexa Flour 647 apoptosis detection kit according to the recommendations of the manufacturer (FCMACS, Nanjing, Jiangsu, China, Cat# FMSAV647-100). PI was also used in order to stain the DNA of the cells.

### Cell culture

THP-1 cells (American Type Culture Collection; ATCC, Manassas, VA, USA) were maintained in RPMI 1640 medium supplemented with 10% (v/v) heat-inactivated FBS and antibiotics (100 units/mL penicillin/streptomycin). The mouse macrophage cell line, RAW264.7, (ATCC) was cultured in DMEM containing 10% heat-inactivated FBS. All the cell types were cultured in a cell incubator (Thermo Scientific 3111, Landsmeer) at 37°C with 5% CO_2_.

### Cell viability assay

Human THP-1 cells (2 × 10^4^ cells/well) were seeded in 96-well plates for the evaluation of all the experimental assays. Following overnight culture, the cells were incubated with various concentrations of FC-99 (0, 1, 10, 50, 100 μM) and/or DMSO for 24 and 48 h. Subsequently, 10 μL CCK-8 was added to each well and incubated for an additional 3 h. The optical density (OD) was measured using a SYNERGY multi-mode reader (BioTek, Winooski, VT, USA) at 450 nm.

### Giemsa staining

Following transfection and treatment with FC-99 and PMA, THP-1 cells were collected and washed two times with PBS and fixed for 5 to 7 min with methanol at room temperature. The cells were air-dried and stained for 15 min with Giemsa stain. The Giemsa solution was prepared according to the manufacturer’s instructions (Merck, Darmstadt, Germany). Subsequently, the cells were washed three times with distilled water, air dried, and observed microscopically following immersion in oil.

### MiRNA transfection

1 × 10^5^ cells/mL were seeded in 6- and/or 12-well plates and transfected with RNA duplexes, 50 nM let-7a-5p miRNA mimic and/or inhibitor (RIBOBIO, Guangzhou, China) by RFect^PM^ small nucleic acid transfection reagent (BAIDAI, Changzhou, China).

### Luciferase reporter gene expression assay

A 1.504 kb 3’-untranslated region (UTR) fragment of BCL-XL containing the putative target site of let-7a-5p was subcloned into the SpeI-SacI sites of the dual luciferase psiCHECK-2 vector (Generay Biotechnology, Shanghai, China). The forward and reverse primer sequences were as follows: 5′- CCACCCTCGAGGCTCCCATGACCATACTGAGGG and 5′- CCACCGCGGCCGCGGATGAGACAGGCCAAGGGTGG, respectively. For mismatch constructs, seven mismatches (underlined in the primers sequence) were introduced in the putative target site with QuikChange1 XL Mutagenesis Kit (Stratagene, La Jolla, CA) according to the manufacturer’s instruction. The primers used for this purpose were the following: forward 5′- GCCCCAGGGTCTTCCGATGGAGAGGCAGGAAGGGCAG and reverse 5′- CTGCCCTTCCTGCCTCTCCATCGGAAGACCCTGGGGC.

THP-1 cells were transfected with 50 nM let-7a-5p miRNA mimic and/or inhibitor using RFect^PM^ small nucleic acid transfection reagent and 800 ng psiCHECK-2 dual-luciferase reporter plasmids. The procedure was conducted according to the Deofect^EU^ Transfection Reagent Protocol (RIBOBIO, Guangzhou, China). At 24 h following transfection, the cells were collected and washed using PBS for 2 times. The cells were mixed well with PLB buffer from the dual-luciferase reporter assay system (Promega, Madison, WI) in order to measure the luciferase activity according to the manufacturer’s instructions.

### RNA isolation and qRT-PCR.

Total RNA from tissues or tumor cells was extracted using Trizol reagent (Invitrogen). Quantitative real-time RT-PCR (qRT-PCR) was conducted, and the expression levels of the genes examined were normalized according to the expression of the housekeeping gene GAPDH. The PCR amplicons obtained with the primer combinations were presented in Table 1.

**Table 1:** Primer sequences for quantitative real-time polymerase chain reaction

Species	Gene		Sequence (5′-3′)
Human	BCL-XL	F^1^ R^2^	GAGCTGGTGGTTGACTTTCTC TCCATCTCCGATTCAGTCCCT
	CD11b	F R	ACTTGCAGTGAGAACACGTATG TCATCCGCCGAAAGTCATGTG
	CD14	F R	CTCTGTCCTTAAAGCGGCTTAC GTTGCGGAGGTTCAAGATGTT
	PBGD	F R	AGCTTGCTCGCATACAGACG AGCTCCTTGGTAAACAGGCTT
	Let-7a-5p	F R	GCGCCTGAGGTAGTAGGTTG CAGTGCAGGGTCCGAGGT
	U6	F R	CTCGCTTCGGCAGCACATATACT ACGCTTCACGAATTTGCGTGTC
Mouse	BCL-XL	F R	GACAAGGAGATGCAGGTATTGG TCCCGTAGAGATCCACAAAAGT
	CD11b	F R	GGCTCCGGTAGCATCAACAA ATCTTGGGCTAGGGTTTCTCT
	CD14	F R	CTCTGTCCTTAAAGCGGCTTAC GTTGCGGAGGTTCAAGATGTT
	TNF-α	F R	CCCTCACACTCAGATCATCTTCT GCTACGACGTGGGCTACAG
	IL-6	F R	TAGTCCTTCCTACCCCAATTTCC TTGGTCCTTAGCCACTCCTTC
	IL-1β	F R	GCAACTGTTCCTGAACTCAACT ATCTTTTGGGGTCCGTCAACT
	iNOS	F R	GTTCTCAGCCCAACAATACAAGA GTGGACGGGTCGATGTCAC
	IL-10	F R	GCTCTTACTGACTGGCATGAG CGCAGCTCTAGGAGCATGTG
	GAPDH	F R	AGGTCGGTGTGAACGGATTTG GGGGTCGTTGATGGCAACA
	PBGD	F R	GTGTTGCACGATCCTGAAACT GTTGCCCATCCTTTATCACTGTA
	Let-7a-5p	F R	GCGCCTGAGGTAGTAGGTTG CAGTGCAGGGTCCGAGGT
	U6	F R	CTCGCTTCGGCAGCACATATACT ACGCTTCACGAATTTGCGTGTC

1. Forward Primers.

2. Reverse Primers.

### Western blot analysis

Western blot was conducted as previously described [30]. The antibodies were used at the following dilutions: mouse anti-tubulin (1:200), mouse anti-caspase3 (1:1,000), mouse anti-BCL-XL (1:1,000), goat anti-mouse IgG (1:10,000) and goat anti-rabbit IgG (1:2,000). The data were shown as fold-values relative to the internal reference control.

### Statistical analysis

The data were expressed as mean ± SEM. The significant differences between the two treatment groups were analyzed using Student’s *t*-test, and the comparison among all four groups was performed using one-way ANOVA. A *P* value of lower than 0.05 (*p* < 0.05) was considered as statistically significant. All statistical analyses were carried out using GraphPad Prism 5 software (GraphPad, San Diego, CA, USA). The statistical comparisons between survival curves were performed using the log-rank (Mantel-Cox) test. The level of significance was set to lower than 0.05 (*P* < 0.05). Each experiment was repeated at least three times.

## SUPPLEMENTARY MATERIALS FIGURES



## Abbreviations

FC-99: N^1^-[(4-methoxy)methyl]-4-methyl-1,2-benzenediamine
PMA: phorbol-12-myristate-13-acetate
CLP: cecal ligation and puncture
AML: acute myelogenous leukemia
DMSO: dimethyl sulfoxide
LPS: lipopolysaccharide
FBS: fetal bovine serum
β-Me: 2-mercaptoethanol
CCK-8: Cell Counting Kit-8
ALT: alanine aminotransferase
AST: aspartate aminotransferase
HE: hematoxylin and eosin
PBS: phosphate-buffered saline
DAPI: 4′,6-diamidino-2-phenylindole
OD: optical density
