# Supplementary material for: FC-99 ameliorates sepsis-induced liver dysfunction by modulating monocyte/macrophage differentiation via Let-7a related monocytes apoptosis
Source: Oncotarget. 2018 Jan 10;9(19):14959–76. doi: 10.18632/oncotarget.24127 (PMC5871089; doi:10.18632/oncotarget.24127)
Supplement: Supplementary file 1 [file oncotarget-09-14959-s001.pdf]

FC-99 ameliorates sepsis-induced liver dysfunction by modulating monocyte/macrophage differentiation via Let-7a related monocytes apoptosis

SUPPLEMENTARY MATERIALS

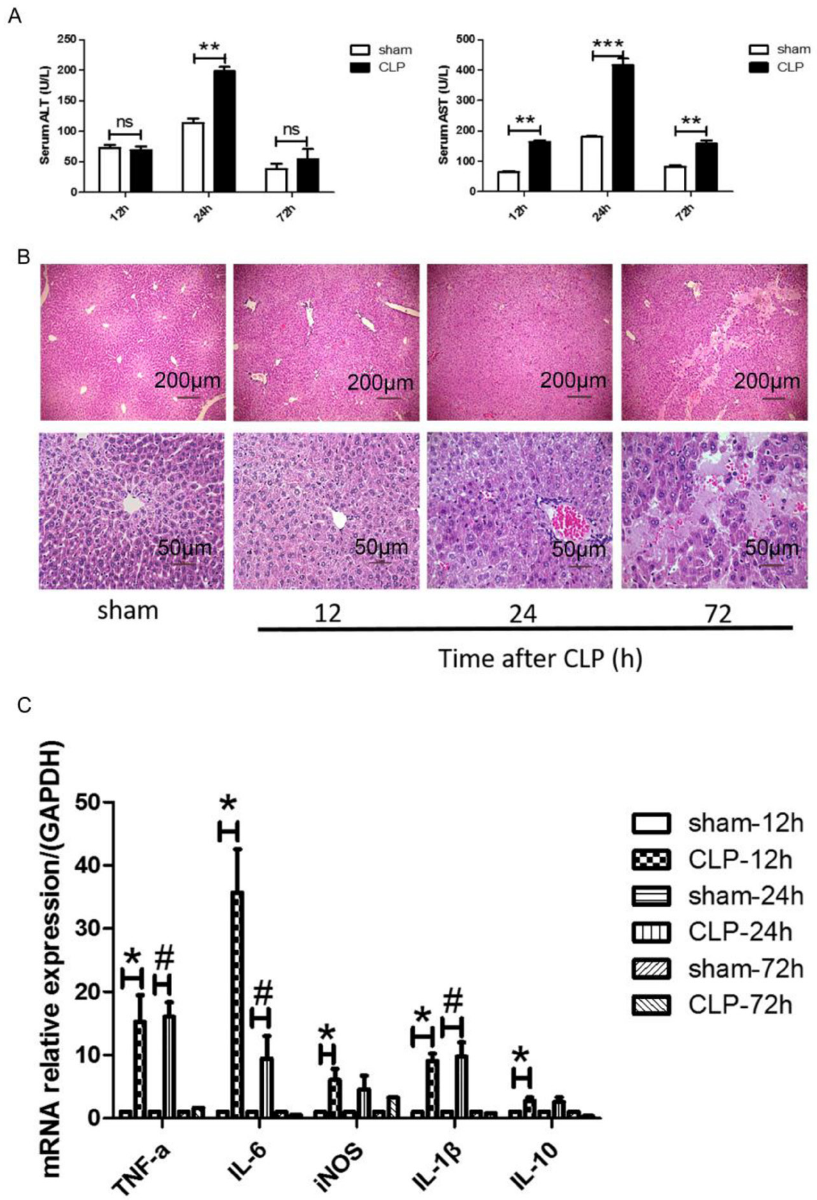

Supplementary Figure 1: Time point selection in CLP model. (**A**) Serum concentrations of ALT and AST from the control group (sham) and the experiment group (12, 24 and 72 h after CLP surgery), (n = 8 mice per group); (**B**) HE staining of liver tissues from the indicated groups, two-dimensional graph. Scale bar, 200 μm or 50 μm (magnification: upper: 100x, under: 400x); (**C**) The mRNA expression levels of the macrophages-associated inflammatory cytokines *IL-6, TNF-α, IL-1β, iNOS* and *IL-10* in the livers of each group of mice were detected by qRT-PCR. $^{*}P < 0.05$, $^{**}P < 0.01$, $^{***}P < 0.005$, vs. sham group; $^{\#}P < 0.05$, $^{\#\#}P < 0.01$, vs. sham-24 h group; ns: P = No significant difference.

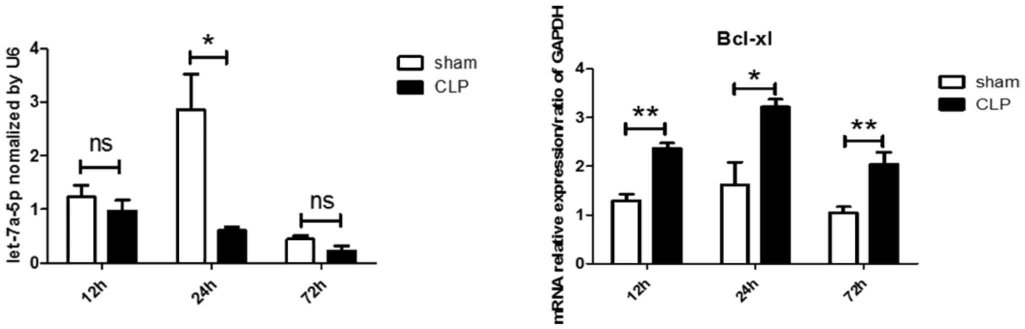

Supplementary Figure 2: Let-7a and its target gene BCL-XL expression in septic livers. qRT-PCR performed to evaluate let-7a and its target gene *BCL-XL* expression in septic livers at 12, 24, 72h in response to CLP or in sham livers. $^{*}P < 0.05$, $^{**}P < 0.01$, $^{***}P < 0.005$, vs. sham group. ns: P = No significant difference.

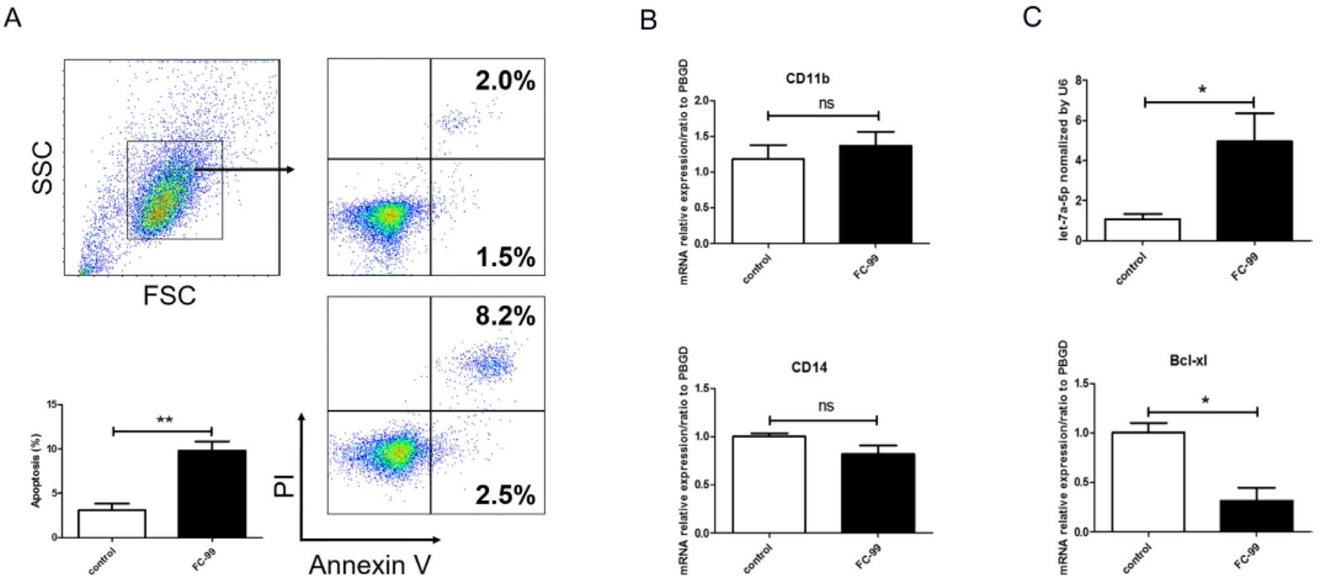

Supplementary Figure 3: FC-99 up-regulates let-7a expression and promotes apoptosis but not differentiation in THP-1 monocytes. (**A**) Annexin V/PI Flow Cytometry results indicated the effect of FC-99 in THP-1 monocytes. (**B**) qRT-PCR applied to detect the expression of macrophages surface markers *CD11b* and *CD14* in FC-99-treated THP-1 without the present of PMA to test the effect of FC-99 in monocytes differentiation. (**C**) qRT-PCR applied to detect the expression of let-7a and the mRNA levels of its target gene *BCL-XL* in FC-99-treated THP-1 without the present of PMA to test the effect of FC-99 in THP-1 monocytes. $^{*}P < 0.05$, $^{**}P < 0.01$, $^{***}P < 0.005$, vs. control group. ns: P = No significant difference.

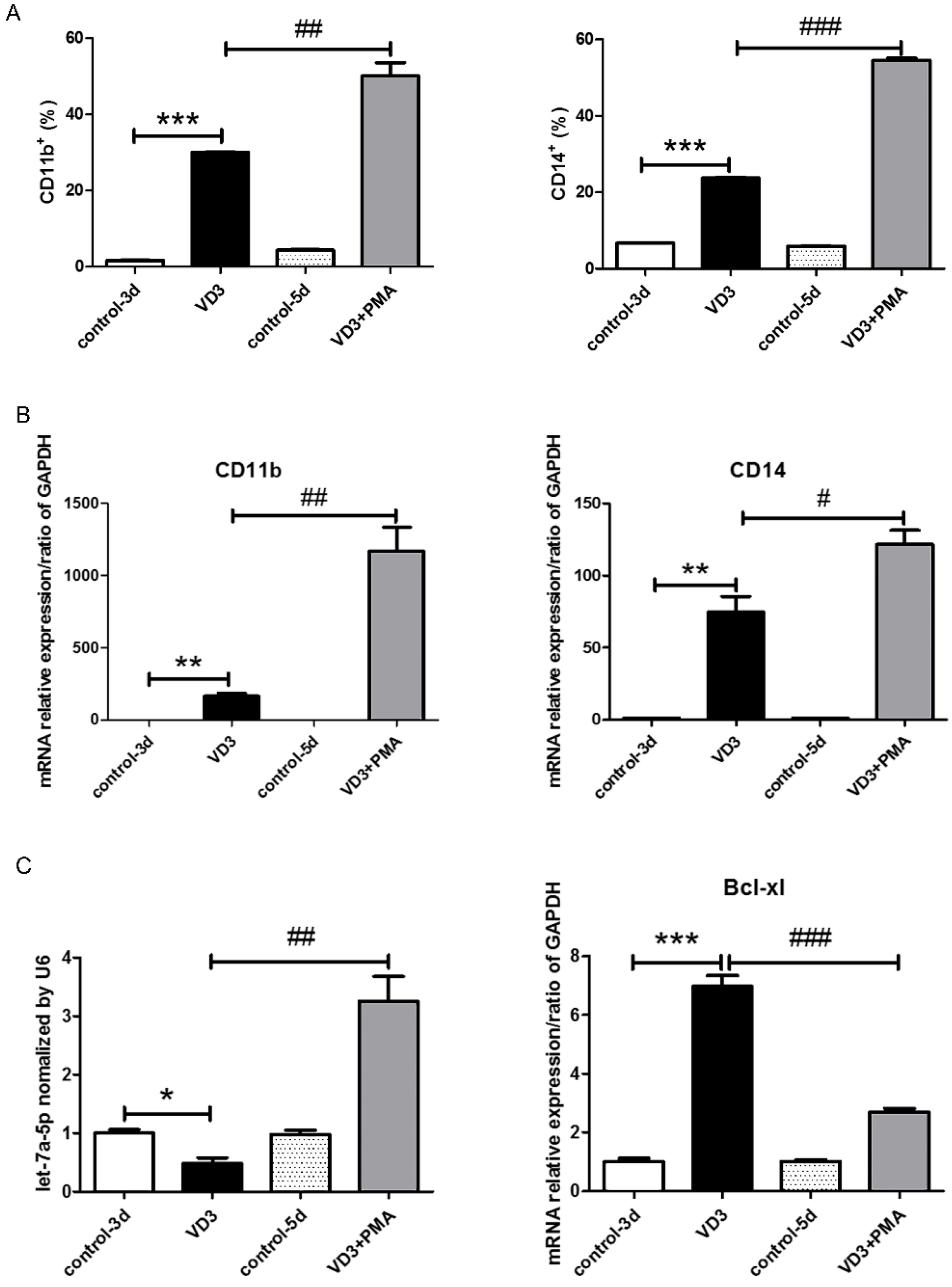

Supplementary Figure 4: Let-7a expression and differentiation associated markers change in VD3-pre-treated HL-60 monocytes. (**A**) All cells were harvested for a Flow Cytometry to detect the differentiation markers, showing the effect of let-7a on the expression of macrophages surface markers CD11b and CD14 under PMA induction for 2d in VD3 (100nM) pre-treated HL-60 monocytes for 3d. (**B**) qRT-PCR showed the mRNA expression of macrophages surface markers CD11b and CD14 in VD3-pre-treated HL-60 monocytes under PMA induction. (**C**) qRT-PCR applied to detect the expression of let-7a and the mRNA levels of its target gene BCL-XL in VD3-pre-treated HL-60 monocytes under PMA induction. $^{*}P < 0.05$, $^{**}P < 0.01$, $^{***}P < 0.005$, vs. control-3d group; $^{\#}P < 0.05$, $^{\#\#}P < 0.01$, vs. VD3 group; ns: P = No significant difference.

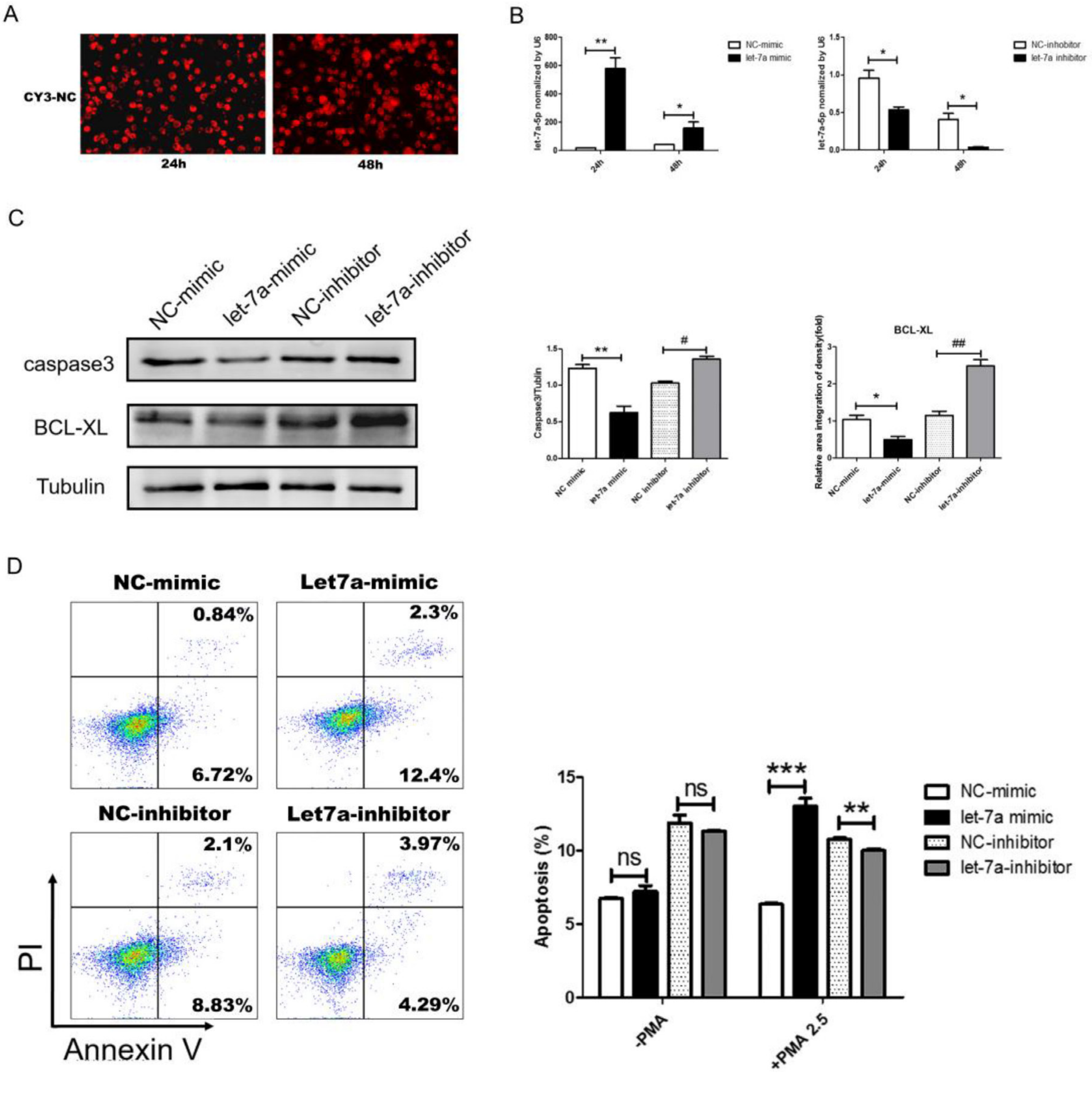

Supplementary Figure 5: Effect of let-7a and FC-99 in PMA-treated THP-1 about apoptosis and differentiation and the effect of PMA in differentiation of monocyte cell line. THP-1 monocytes were transfected with let-7a-5p mimic, let-7a-5p inhibitor or NC-mimic, NC-inhibitor as a control for 24h, and then incubated with or without PMA for another 48h. (**A**) Photograph taken with optical microscope (original magnification, ×200) shows the transient transfection efficiency of CY3 conjunct-negative control mimic/ inhibitor in THP-1 monocytes for 24h and 48h. (**B**) qRT-PCR applied to detect the expression of let-7a and the mRNA levels of its target gene *BCL-XL* in THP-1 transfected for 24 and 48h to further detect the transfect efficiency. (**C**) Western blot reveals the levels of apoptotic marker proteins caspase3 and anti-apoptosis protein BCL-XL. (**D**) Annexin V/PI Flow Cytometry results indicated the effect of let-7a in THP-1 apoptosis during differentiation induced by PMA. Histogram shows the ratio of apoptosis cells. $^{*}P < 0.05$, $^{**}P < 0.01$, $^{***}P < 0.005$, vs. NC-mimic group; $^{\#}P < 0.05$, $^{\#\#}P < 0.01$, vs. NC-inhibitor group; ns: P = No significant difference.
